# Detecting Incremental Frequent Subgraph Patterns in IoT Environments

**DOI:** 10.3390/s18114020

**Published:** 2018-11-18

**Authors:** Kyoungsoo Bok, Jaeyun Jeong, Dojin Choi, Jaesoo Yoo

**Affiliations:** Department of Information and Communication Engineering, Chungbuk National University, Chungdae-ro 1, Seowon-Gu, Cheongju, Chungbuk 28644, Korea; ksbok@chungbuk.ac.kr (K.B.); jjjeong@chungbuk.ac.kr (J.J.); mycdj91@gmail.com (D.C.)

**Keywords:** graph stream, IoT, subgraph pattern, frequent pattern detection, incremental

## Abstract

As graph stream data are continuously generated in Internet of Things (IoT) environments, many studies on the detection and analysis of changes in graphs have been conducted. In this paper, we propose a method that incrementally detects frequent subgraph patterns by using frequent subgraph pattern information generated in previous sliding window. To reduce the computation cost for subgraph patterns that occur consecutively in a graph stream, the proposed method determines whether subgraph patterns occur within a sliding window. In addition, subgraph patterns that are more meaningful can be detected by recognizing only the patterns that are connected to each other via edges as one pattern. In order to prove the superiority of the proposed method, various performance evaluations were conducted.

## 1. Introduction

A graph is a data structure consisting of vertices and edges connecting the vertices. These graphs have been used to represent many-to-many relationships between objects, where a vertex represents an object, and an edge represents a relationship between objects [1,2,3,4]. Graphs are widely used in various fields, such as road networks, bioinformatics, and social networks [5,6,7]. For example, in a traffic network, regions are represented by vertices, and roads are expressed by edges. In a social network, vertices represent users, and edges express the relationships of followers and friends. In bioinformatics, graphs are used to model interactions between biomolecules, and graphical frequent pattern detection is used for protein function prediction, mutant gene discrimination, disease type identification, and so on [8,9,10]. Now, graph data change in real time due to the activation of Internet of Things (IoT) along with the advances in network technologies. Stream data in which vertices and edges that make up a graph continuously change are called graph streams. Graph streams have been used in various fields for different applications, such as abnormal detection, real-time trend analysis, and event detection [11,12,13,14,15,16]. As the graph stream has been applied in various fields, a large number of studies on various techniques for the analysis of graph streams have been conducted [17,18,19,20].

As the vertices and edges are continually added, deleted, and updated in IoT environments, studies on the detection or analysis of changes in graphs have also been conducted. Various approaches, such as graph clustering, graph stream classification, subgraph mining, and frequent subgraph pattern detection, have been proposed for graph analysis [5,21,22,23,24,25,26,27]. Frequent subgraph pattern, which detects a subgraph frequently occurring during a specific period is a widely used analysis method for graph streams [28,29,30,31,32]. In the IoT environment, frequent subgraph pattern is used for analyzing interactions among various objects or for determining anomalies [33,34,35]. For example, in anomaly detection, the process of data transmission between IoT devices is modeled as graph data, and then a frequent subgraph pattern is generated. If an infrequent subgraph pattern occurs, it is determined as an anomaly.

The frequent subgraph detection in static graphs identifies frequently occurring subgraphs in the whole graph. However, graph streams continue to change vertices or edges over time. A graph matching finds a correspondence between the vertices and the edges of two graphs that satisfies some constraints. Since there are various subgraph patterns that can occur in the graph stream, all possible subgraphs should be compared when using graph matching. Therefore, it takes a lot of comparison time to detect frequent subgraphs by using graph matching in the graph stream. As the utilization of frequent subgraph pattern detection in recent graph streams has increased, various approaches have been actively conducted [29,36,37,38] in order to deal with this. In Reference [30], Data Stream Tree (DSTree) was proposed to store graph streams in memory efficiently during frequent subgraph pattern detection. Once the graph stream data are inputted, a DSTree is constructed, using whichever FP-tree was constructed to detect the frequent pattern. In Reference [31], Data Stream Matrix (DSMatrix) was proposed for storing graphs more efficiently than the DSTree proposed in Reference [30]. The DSMatrix is a two-dimensional array, so it can be constructed at a lower cost than that of DSTree. In addition, the frequency level of each pattern is calculated with a depth-first search after constructing a new FP-tree. In Reference [32], a frequent detection that considers connectivity was proposed. In this method, a simple frequent subgraph pattern detection using the AND operation was used, and only connected patterns are detected by managing adjacent edge information. However, the existing methods have several problems. First, Reference [30] uses many pointers because it constructs a DSTree, which is time-consuming. In addition, References [30,31] detect insignificant frequent patterns since these methods do not consider connectivity. Furthermore, References [30,31,32] employs a sliding window to detect all frequent patterns. However, since they do not process duplicate operations, their operation time is degraded.

Real-time processing is required to detect frequent subgraphs in the graph stream. Real-time processing of graph streams needs to reduce processing time. Since real-time processing techniques use memory, it is necessary to minimize memory usage. In this paper, we propose a new method that can detect a frequent subgraph pattern incrementally in graph stream data inputted in real time. The proposed method models the relationship between IoT devices in the IoT environment as a graph and detects frequently occurring subgraphs above the threshold value as vertices and edges constantly change over time. Frequent subgraphs that are detected through the proposed method are used to determine anomalies in the IoT environment or to analyze interactions among IoT devices. The proposed method is aimed at reducing processing time and memory usage to process graph streams in real time. The proposed method constructs DSMatrix for graph streams and does not construct FP-tree to reduce the memory usage. The proposed method determines whether re-calculation is needed after calculating whether a subgraph pattern detected from the previous sliding window will be frequent or not in the future. By doing so, only necessary calculations are performed, thereby reducing the total computation. In addition, more meaningful patterns can be detected by recognizing the patterns that are connected through edges between the patterns as one pattern. In addition, more meaningful patterns can be detected by recognizing patterns that are connected to each other through their edges as one pattern.

This paper is organized as follows. Section 2 analyzes the existing methods that detect a frequent subgraph pattern in graphs and presents the limitations of the previous studies. Section 3 explains the proposed frequent subgraph pattern detection method, and Section 4 presents performance evaluation results that verify the superiority of the proposed method. Finally, Section 5 describes the conclusions of this study and future research.

## 2. Related Works

In Reference [30], a frequent subgraph detection was proposed in which graph streams are rapidly stored in memory using a tree structure called DSTree. Once the graph streams are inputted, each edge is arranged in order, and then a tree is constructed. Since each of the nodes in a DSTree maintains edge information by batch, it can be maintained by just changing the relevant information, even if a window slide has moved. Each node in a DSTree stores the names of the graph edges and the count of the appearances for each batch, each of which is divided by a semicolon. If there is a scarce graph, a tree is constructed only with regard to the edges newly created. Thus, efficient graph storage can be achieved. 

In Reference [31], a DSMatrix, which can store graph streams more efficiently than the previous approaches were proposed. A DSMatrix is a 2D structure that represents whether an edge in the graph is generated by one or zero to store graph stream data in a small memory usage. To detect a frequent pattern, two approaches are used: Recursive-FP-tree and FP-trees for only frequent singletons. Using the recursive-FP-tree method, graph streams are stored in the DSMatrix and then an FP-tree is constructed for all edges. All frequent patterns are detectable by recursively constructing the FP-tree. This method can easily extract preferred subgraph by constructing multiple FP-trees. In the FP-trees for only frequent singletons, an FP-tree is first constructed with regard to all edges, and then each node is visited using the depth-first search method to calculate the number of occurrences of frequent patterns. This method can detect frequent patterns at a lower cost than that of the recursive-FP-tree since FP-trees are constructed for only a singleton edge. 

In Reference [32], a frequent subgraph pattern using a simple AND operation without FP-tree was proposed. This method detects meaningful frequent patterns considering the connectivity between edges. It uses two approaches: one excludes unconnected patterns after generating candidates of frequent patterns using the AND operation for frequent subgraph pattern detection, and the other verifies whether the connection is made prior to starting the AND operation, and then it performs the operation. 

A DSTree can store data in memory efficiently during frequent subgraph pattern detection [30]. Once graph stream data are inputted, a DSTree is constructed using whichever FP-tree was constructed to detect the frequent pattern. A DSMatrix can store graphs efficiently [31]. The DSMatrix is a two-dimensional array, so it can be constructed at a lower cost than that of the DSTree. In addition, a frequency level of each pattern is calculated with a Depth-First Search (DFS) after constructing a new FP-tree. In Reference [32], a frequent detection method that considers connectivity was proposed. In this method, a simple frequent subgraph pattern detection using the AND operation is used, and only connected patterns are detected by managing adjacent edge information in a table. However, the existing methods have some limitations as Table 1. Reference [30] has a shortcoming when a graph’s structure changes significantly because the structure of the DSTree also changes, which takes a lot of time to reconstruct. In addition, when a DSTree is constructed, a large number of pointers are used when graph data are dense, entailing a large management cost to maintain the tree. To solve this problem effectively, a DSMatrix was proposed [31]. However, it requires many operations and a lot of memory because a large number of FP-trees are constructed during frequent subgraph pattern detection. It requires a lot of computation time because it travels all over the FP-trees to generate frequent patterns. Reference [32] uses AND operation to detect frequent patterns. However, it has a problem with performing comparative operations on the edges that are not likely to be detected in the future. In addition, References [31,32] do not solve the duplicate calculation problem, which is one of the drawbacks when using a sliding window, thereby degrading the performance.

In this paper, we propose an incremental frequent pattern detection to solve the problems of existing methods. The proposed method reduces the amount of computation by reusing the results of analysis from the previous sliding window as the window slide moves. It stores the input graph streams in DSMatrix to reduce the cost of building a DSTree according to the changes of the graph stream. It reduces memory usage because it only manages previously detected frequent pattern information. The detected patterns are calculated and managed separately for the next few sliding windows. This calculated value reduces the overall computation because the next sliding window only performs AND operations on subgraph patterns that are likely to occur in a frequent pattern. It also reduces unnecessary comparison operations because only the connections between patterns are determined in one pattern. 

## 3. The Proposed Frequent Subgraph Pattern Detection

### 3.1. Preliminary

A graph is a data structure to express a multiple-relationship among objects. The graph consists of the vertex representing the object and the edge representing the relationship among the objects. Definition 1 represents the definition for the graph. If data transmission between IoT devices is represented as a graph in the IoT environment, a vertex represents IoT device and an edge represents data transmission status between IoT devices. Similarly, when a human network on a social network is represented as a graph, the vertex is the user and the edge is the friendship. Graphs are divided into directed and undirected graphs. The undirected graph does not take into account the direction of the edge between the vertices, but the directed graph displays the arrow lines along the connected direction between the vertices. When the vertices or edges that make up the graph change continuously over time, they are called graph streams. Graph streams are graphs that vertices and edges change dynamically over time. The graph streams occur frequently in IoT environments where the relationships of objects change. Definition 2 defines the graph stream $G_{t}$ that occurs in time t.

**Definition** **1.** *Graph* G

*Given a set of vertices*V*and a set of edges*E*which are subsets of*V×V*, a graph is defined as an order pair*G=(V,E).

**Definition** **2.** *Graph stream* $G_{t}$

*Given a set of vertices*$V_{t}$*at time*t*and a set of edges*$E_{t}$*which are subsets of*$V_{t}\times V_{t}$*at time t, streamgraph is defined as order pair*$G_{t}=(V_{t},E_{t})$.

In a static graph with no change, a frequent subgraph represents a subgraph that appears above the threshold within the given graph. Since vertices and edges change continuously in the graph stream over time, a frequent subgraph is defined as a subgraph with more than a threshold within a continuous time interval, as defined in Definition 3.

**Definition** **3.** *Frequent subgraph in graph stream*

*Given a stream graph, frequent subraph is defined as*Freq(SG)≥θ*, where*SG*is a subgraph,*Freq(SG)*is the occurrence percentage of graph that contain*SG.

We incrementally detect a frequent subgraph in the graph stream. It constructs the DSMatrix proposed in Reference [31] to determine whether or not each edge occurs in the input graph stream. With a two-dimensional array called DSMatrix, it is possible to store a large amount of edge information in a small space. The proposed method generates subgraphs if neighboring edges occur frequently and manage their occurrence information in the frequent subgraph management table by using DSMatrix. It determines incrementally whether a frequent subgraph has occurred by using FiB, FiS, and slideNum in the frequent subgraph management table. Table 2 shows the notations used in the proposed method.

### 3.2. Overall Procedure 

As graph streams have been applied to IoT, including anomaly detection, real-time trend analysis, and event detection, a large number of studies have been performed on various methods that analyze graph streams [39,40]. There are three considerations when a frequent subgraph pattern is detected in graph stream data. First, frequent subgraph pattern detection should be fast and should utilize limited storage space efficiently. Since stream data are supplied constantly and no end point is specified, the graph data to be analyzed change in real time. Thus, when graph are inputted and analyzed, graph should be deleted, to some extent, to ensure sufficient memory for analyzing the next input graph. Second, input graph differ as time passes. That is, the currently frequent patterns may not continue for the next input graph, and vice versa. Finally, patterns should be generated considering the connectivity of graphs. Here, connectivity means that detected patterns are connected to one another. 

In this paper, we propose a frequent subgraph pattern detection incrementally for graph stream data in IoT. The proposed method determines a frequent pattern for performing anomaly detection or for analyzing the cooperative relationship between IoT devices. For example, anomaly detection is determined as an anomaly when a subgraph that is not detected as a frequent pattern occurs, or cooperative relationship analysis determines that mutual cooperation is very high in the event of a frequent pattern among IoT devices. A frequent subgraph pattern is a connected subgraph that occurs above a threshold for a particular time interval. The proposed method reuses the analyzed results from the previous window when the window is moved to reduce the amount of computation. It stores the input graph streams in a DSMatrix; then, frequent patterns can be detected through the simple AND operation. Here, the detected patterns are calculated for determining whether they are frequent or infrequent in the several sliding windows in the future, and then managed in a table separately. Through the calculated values, only necessary calculations are performed in the next sliding window to reduce the overall computation. 

**Figure 1:**
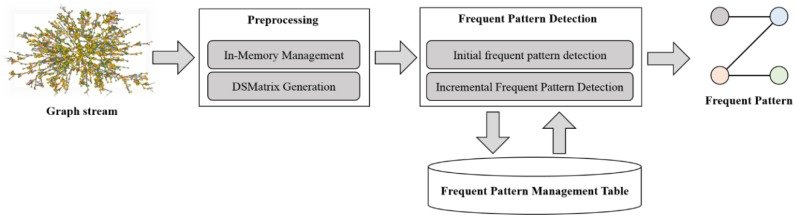
Overall processing procedure.

The overall processing procedure of the proposed method is shown in Figure 1. The preprocessing efficiently stores the input graph in the memory and generates a DSMatrix. Here, a DSMatrix can store a large amount of graph data in a small space using a 2D structure. During frequent subgraph pattern detection, an operation to generate the actual frequent patterns is performed. When graph patterns are detected, graph pattern occurrences are summed to check whether frequent patterns occur, and the occurrences of two patterns are summed again via the AND operation, thereby enabling frequent subgraph pattern detection consisting of multiple edges. Here, the information on the frequent patterns generated from the previous window slide is employed through the use of a frequent subgraph pattern management table. In the frequent subgraph pattern management table, whether the previously detected patterns will be frequent or infrequent in the next several sliding windows is calculated and stored; through this value, only necessary calculations are performed in the next sliding window, thereby reducing the overall computation. Finally, the detected frequent patterns are delivered to the user and are simultaneously stored in the frequent subgraph pattern management table to be utilized in the next window slide.

### 3.3. Preprocessing 

Preprocessing stores graph streams in the memory and configures the DSMatrix. Since graph streams are continuously generated, graphs should be analyzed and deleted to ensure sufficient memory for processing the next input graph. In addition, all patterns can be generated for frequent patterns, considering not only currently generated graph but also previous and future graph. We use a 2D array structure called a DSMatrix in the preprocessing considering this characteristic. Since DSMatrix is a Boolean-type array, it can store more data in a smaller space than the DSTree uses in the existing method [30]. In addition, since the presence of an edge in a graph is expressed by one or zero, data can be added and deleted rapidly, which is suitable for the sliding window. 

**Figure 2:**
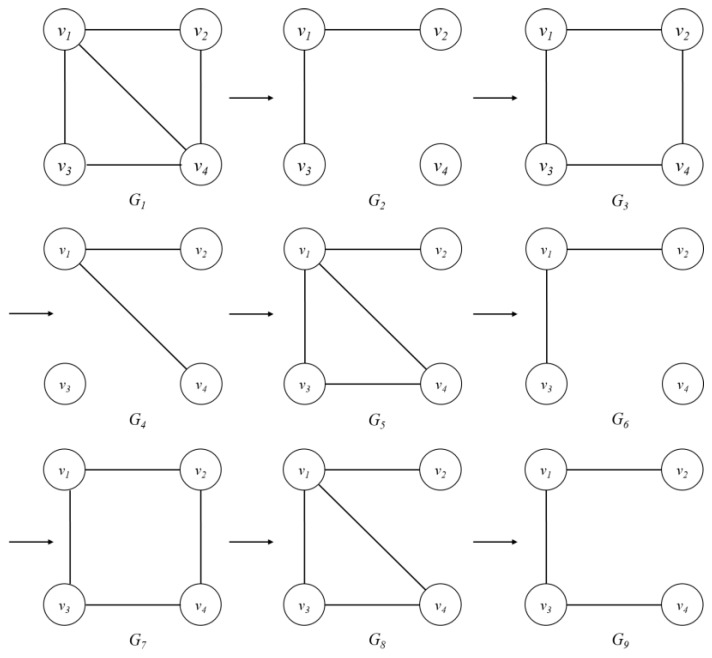
Example of a graph stream, where *G_t_* is a graph at time *t*.

In a DSMatrix, a single window slide consists of the number of batches, which is set by the user. Table 3 shows the DSMatrix that results when the graph streams $G_{1}\sim G_{9}$ are inputted, as shown in Figure 2. Here, one batch consists of three graphs, and three batches comprise a single sliding window. Each edge is represented by the names of the two connecting vertices. For example, the edge connected by vertices $v_{i}$ and $v_{j}$ is expressed as $<v_{i},v_{j}>$. With the edges expressed in this way, the contents parts in Table 3 represent whether the edges occur or not as one or zero, respectively.

### 3.4. Initial Frequent Subgraph Pattern Detection

The initial frequent subgraph pattern detection generates a frequent subgraph pattern as a reference pattern during the incremental frequent subgraph pattern detection. We considers two features when a frequency pattern is detected. First, since stream data are continuously inputted, graphs should be analyzed and deleted, to some extent, when inputted to ensure sufficient memory for analyzing the next input graph. This requires a technique that detects a frequent subgraph pattern rapidly. The pattern occurrences are summed to check whether frequent patterns occur, and the occurrences of two patterns are summed again via the AND operation, thereby detecting frequent patterns consisting of multiple edges continuously. Here, since the operation that sums the number of occurrences and the AND operation are simple, patterns can be generated quickly. Second, we consider connectivity. If two patterns are far away from each other, it is difficult to see these two patterns as a single pattern, even if they occur simultaneously. Thus, our method detects more meaningful frequent patterns by considering connectivity.

A frequency level of a single edge is checked first to detect a frequent pattern. The number of occurrences in a single batch per edge in the DSMatrix, which is made in the preprocessing, is calculated and inputted to the Frequency in Batch (FiB) column in the frequent subgraph pattern management table, as presented in Table 4. FiB refers to the number of occurrences of the corresponding edge in a single batch. In addition, Frequency in Sliding window (FiS) refers to a value that sums all FiBs calculated previously, by which the determination of whether the current edge is frequent in the current sliding window can be verified. For example, the edge $<v_{1},v_{2}>$ occurred three times in every single batch and occurred a total of nine times in the current window slide.

slideNum is a calculated value determining whether a single edge will be frequent or not during the next several slides. For example, even if zero is inputted to all $<v_{1},v_{2}>$ in the next window slide, the frequency count is still six, which is a frequent edge. However, if zero is input to all $<v_{1},v_{2}>$ again in the next window slide, the frequency count is three, which is not a frequent edge. Thus, slideNum becomes one. The algorithm to calculate slideNum is shown in Algorithm 1. slideNum is calculated as FiS and is divided into two cases, i.e., when it is larger or smaller than the threshold. If it is larger than the threshold, the remaining number, after removing the first batch from the current sliding window, is calculated as batchCount, and it is then determined whether batchCount exceeds the threshold, assuming that all new input batches are zero. This process is iterated until batchCount becomes smaller than the threshold. Then, the result is returned. If FiS is smaller than the threshold, whether batchCount exceeds the threshold is checked, assuming that the next input batches are all one, and the count is returned.

**Algorithm 1.** Algorithm to calculate slideNum.
*Input:*

*FiB[] – array of FiB, which is the number of edges in a batch*

*FiS - The number of edges in sliding window*

*th - threshold*

*Output: slideNum*

*slideNum ← 0*

*if FiS >= th then*
 *batchCount ← FiS–FiB[0]*
 *while batchCount >= th and slideNum < slidingWindowSize do*  *batchCount ← batchCount–FiB[slideNum+1]*  *slideNum ← slideNum+1* *return slideNum*
*else*
 *batchCount ← FiS–FiB[0]+batchSize* *while batchCount < th and slideNum < slidingWindowSize do*  *batchCount ← batchCount–FiB[SlideNum+1] + BatchSize*  *slideNum ← slideNum + 1* *return -slideNum*


After single edges, which are frequent, are all detected, patterns consisting of multiple edges are detected. If the AND operation is applied to the detected two patterns, it can identify the number of frequent occurrences of two patterns at the same time. A pattern is expanded continuously using this process. That is, if a pattern consisting of two edges is detected, a single frequent edge is employed. In the previous example, if the AND operation is applied to $<v_{1},v_{2}>$ and $<v_{1},v_{3}>$ among the single edge patterns, it produces 111;011;111 so that the number of occurrences becomes eight, which indicates that $<v_{1},v_{2},v_{3}>$ is also a frequent pattern.

Additionally, when detecting patterns consisting of multiple edges, connectivity should be taken into consideration. An edge name is used to determine whether two patterns are connected. That is, if there is a duplicate vertex ID in the pattern’s name, it indicates that the two patterns are connected. If two patterns are not connected, they are not included in the frequent patterns. That is, as $<v_{1},v_{2}>$ and $<v_{3},v_{4}>$ have no duplicate vertex ID, the AND operation is not performed. The detected frequent patterns are stored in the frequent subgraph pattern management table, as presented in Table 5. Here, FiB, FiS, and slideNum have the same as those used for the single edge.

### 3.5. Incremental Frequent Subgraph Pattern Detection

The incremental frequent subgraph pattern detection is a technique to resolve duplicate calculations, which is a problem in the sliding window. Since graph are duplicated in sliding windows, performance is degraded. To resolve this problem, we store previously detected frequent subgraph pattern information in the frequent subgraph pattern management table and generate a new frequent pattern using the stored frequent pattern information. In the frequent subgraph pattern management table, whether the previously detected patterns will be frequent or infrequent in the next several sliding windows is calculated and stored; through this value, only necessary calculation is performed in the next sliding window, thereby reducing the overall computation cost.

**Figure 3:**
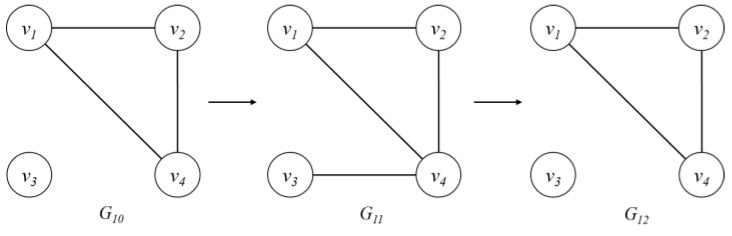
New input graph stream, where *G_10_* is a graph at time 10, *G_11_* is a graph at time 11, and *G_12_* is a graph at time 12.

When new graphs $G_{10}\sim G_{12}$ are inputted as shown in Figure 3, a new batch is added to the DSMatrix. If the new batch is added, FiB and FiS are calculated and stored, and the same is done in the basic frequent subgraph pattern detection. However, if slideNum is not zero, slideNum is decreased and FiB and FiS are not calculated. That is, $<v_{1},v_{2}>$, $<v_{1},v_{3}>$, and $<v_{2},v_{4}>$ do not calculate FiB and FiS, as presented in Table 6.

When detecting frequent patterns consisting of multiple edges, a similar approach to the previous procedure is used. In Table 7, pattern $<v_{1},v_{2},v_{3}>$ has one slideNum so that it can be identified as a frequent subgraph pattern without the need to calculate FiB and FiS. Thus, after slideNum is modified to zero, a frequent subgraph pattern is maintained. For the pattern $<v_{1},v_{3},v_{4}>$, its slideNum is zero. Thus, FiB and FiS are calculated by applying the AND operation only to the new input batch of $<v_{1},v_{3}>$ and $<v_{3},v_{4}>$. That is, since $<v_{1},v_{3}>$ is 000 and $<v_{3},v_{4}>$ is 010, 000 & 010 = 000. Thus, it becomes FiB = 0 and FiS = 3 for the newly added part. Here, if FiS is larger than the threshold, slideNum is calculated.

## 4. Performance Evaluation

To prove the superiority of the proposed method, performance evaluation was conducted by comparing it with the existing methods [31,32]. For convenience, the method proposed in Reference [31] is called DSMatrix and the method proposed in Reference [32] is called SAND. Table 8 summarizes the performance evaluation environment. The performance evaluation program was implemented using Java. For performance data, arbitrarily created graph stream data and real data were used. For real data, as-Caida [41], one of the datasets provided by SNAP [42], was employed. The data were graphs that represent a connection relationship with network routers stored on a time basis. The data consist of 65,003 vertices and 30,000 edges. A total of 122 datasets were inputted according to the time sequence. The performance evaluation compared the processing time when the batch size, window slide size, and threshold value were changed. If the frequent subgraph pattern detection time is slower than the stream input rate, frequent patterns that cannot be discovered may be found. Thus, the accuracy of the frequent subgraph pattern detection results can be evaluated. Finally, the amount of memory used in the frequent subgraph pattern detection process was measured to evaluate the space efficiency of DSMatrix. 

**Figure 4:**
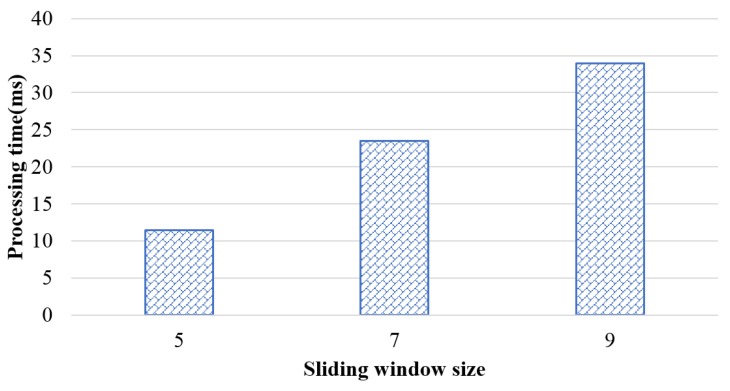
Processing time of the proposed method according to sliding window size.

When a frequent subgraph pattern is detected, if a graph stream input rate is faster than the processing time, a frequent subgraph pattern may occur in which a new graph may be lost before being detected. Thus, this study measured the frequent subgraph pattern detection time per sliding window to establish the right input rate. Figure 4 shows how much time is consumed in each window slide to verify whether data are lost. A total of 122 datasets were inputted according to the time sequence. A single batch consisted of 10 graphs, and the processing time according to the sliding window size was evaluated. The performance evaluation results showed that the frequent subgraph pattern detection time in each sliding window was calculated by measuring the processing time for each sliding window and taking the mean value. When a single sliding window consists of five batches, the processing time is approximately 11 ms. However, if an interval of the graph input is smaller than 11 ms, frequent patterns may be lost. Thus, frequent patterns can be detected accurately when the data size, sliding window size, and batch size are appropriately selected according to the data characteristics in application fields.

**Figure 5:**
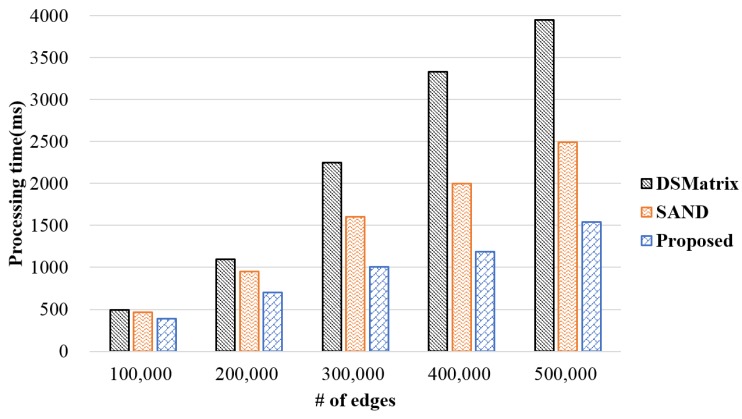
Processing time according to the number of edges.

Generally, as graph size increases, the rate of frequent subgraph pattern detection increases. As a result, if too much time is taken to detect a frequent subgraph pattern from a large number of graphs, the significance of the detected frequent subgraph pattern may be weakened. Figure 5 shows the comparison of the experimental results for data processing time between the proposed and existing methods as the number of edges increased. This experiment generated arbitrary graphs and changed the number of edges in the graphs to conduct performance evaluation. A batch consisted of 100 graphs, and a window slide consisted of five batches. In addition, a threshold was set to 80% to detect patterns that appeared more than 400 times in the window slide. In this experiment, when the number of edges was small, no significant processing time was revealed. However, when the number of edges was increased, the processing time in the proposed method was reduced by up to 60% compared to that of the existing methods. The reason for this was because duplicate processing results also increased as the number of edges increased, and the existing methods performed duplicate processing continuously.

**Figure 6:**
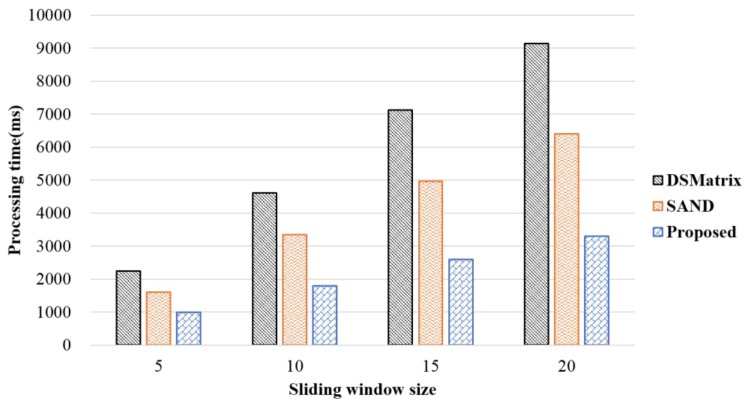
Processing time according to the sliding window size.

DSMatrix is based on sliding windows. Accordingly, its processing time depends on the sliding window size. Thus, it is important to set a sliding window size that is suitable for different applications. Figure 6 shows the difference in processing time between the proposed and two existing methods according to the window slide size. Each graph is an arbitrarily created graph that consists of 300,000 edges. In the figure, when 20 batches are included in a single sliding window, the proposed method reduces the processing time by 63% compared to that of DSMatrix and by 50% compared to that of SAND. This result verifies that, compared to the existing methods, the performance of the proposed method improves as the number of sliding windows increases. This is because, during several graph streams, according to the value of slideNum, it was determined that no calculation will be needed in the future when using the incremental frequent subgraph pattern detection method. As a result, the overall computation cost was reduced, as patterns not needing calculation increased when the sliding window size increased.

**Figure 7:**
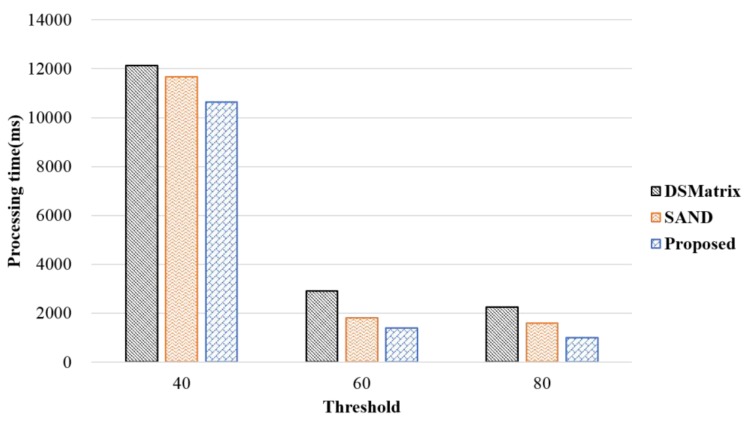
Processing time according to threshold.

A frequent subgraph pattern means a subgraph that frequently occurs above the threshold. We confirmed through various experiments that the results of frequent pattern detection of the proposed method are identical to those of the existing methods. Therefore, we compare the proposed method with the existing methods in terms of the processing time according to the changing threshold. Figure 7 shows the processing time according to a threshold value. The graph in this performance evaluation used arbitrarily generated data, which consisted of 300,000 edges; one batch consisted of 100 graphs, and one sliding window consisted of five batches. As shown in the figure, the processing time slowed down rapidly as the threshold became smaller. This was because the number of cases to be considered when two or more frequent patterns were detected increased exponentially. However, when the threshold was set to 80%, the processing time in the proposed method was faster: 60% of that of DSMatrix and 55% of that of SAND. The reason for this is, as the threshold value became larger, slideNum was likely to increase, which improved the performance. Thus, it is important to select a threshold value that is suitable to the data and the application field.

**Figure 8:**
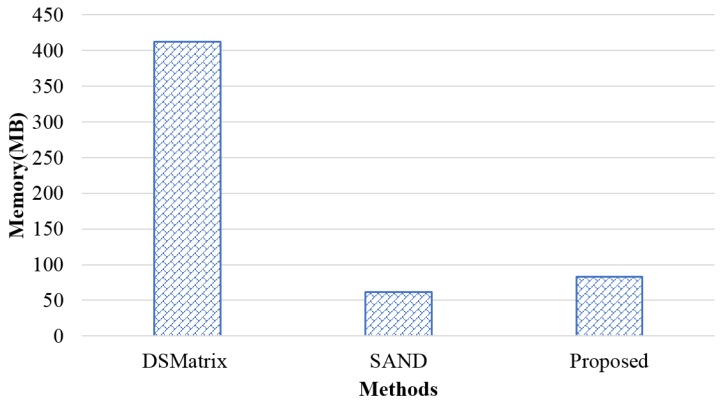
Memory usage.

A larger memory usage is advantageous to the frequent subgraph pattern detection of larger graph sizes. Frequent patterns may occupy more space than graph increases occupy. Thus, if the memory usage is bigger, frequent patterns of larger data can be detected. Figure 8 shows the memory usage measured using the as-Caida data. Here, the sliding window size was five, and each batch consisted of 10 graphs. The proposed method used a memory of 83 MB on average, which used more memory than SAND (62 MB on average). This was because the proposed method employs the frequent subgraph pattern management table to improve the processing time, resulting in additional memory space for pattern management. In addition, DSMatrix used 412 MB of memory on average because it employs many pointers to construct trees, and there are many duplicate data because trees are constructed for each edge. However, as the proposed method and SAND detect frequent patterns using a bit product rather than a tree, they used less memory.

The existing methods require high computation costs to detect frequent patterns after the initial graph is generated. In addition, the detection of frequent subgraphs using sliding windows resulted in unnecessary comparison. The proposed method reduces processing time because it manages frequent subgraph patterns and performs comparative operations only on subgraph patterns that may occur in the future. In addition, similar to the method proposed in Reference [32], only subgraphs managed in frequent subgraph patterns perform AND operation, thereby reducing the comparison operation. The proposed method reduces the number of edges, the size of the sliding window, and the processing time as the threshold changes. Memory based processing is performed because real-time processing is required to detect frequent subgraph patterns in the graph stream. Therefore, it is important to reduce memory usage when detecting frequent subgraphs. DSMatrix [31] uses a lot of memory because it constructs DSMatrix for input graph streams and constructs the FP-Trees for frequent pattern detection. SAND [32] uses the least memory since it does not deploy FP-Trees and only constructs DSMatrix for input graph streams. The proposed method constructs DSMatrix for input graph streams, similar to SAND, but additionally manages the frequent subgraph patterns to reduce unnecessary comparison operations. Therefore, it uses more memory than SAND. However, the proposed method does not use that much memory since it only manages frequently occurring subgraphs, not all subgraphs.

## 5. Conclusions

In this paper, we proposed an incremental processing method to detect frequent patterns from graph streams. The proposed method can reduce the processing time by managing frequent patterns discovered in previous sliding windows in a frequent subgraph pattern management table, then utilizing the data in the table for the next sliding window. It also generates more meaningful frequent patterns by considering connectivity. The performance evaluation results verified that the proposed method could reduce duplicate operations, which was an important feature since the amount of duplicated data increased in the sliding windows when the graph and sliding window sizes increased. As a result, the processing time was reduced by 55% on average, compared to the existing methods. The proposed method manages frequent patterns in the table, so it has the limitation of needing excessive memory space to manage frequent patterns in the table, and more time is needed to scan them as the number of frequent patterns increases. Thus, for future research, a study will be conducted using an index for direct access to the required pattern to reduce the cost of scanning when the number of patterns to be managed increases. 

## Figures and Tables

**Table 1:** Characteristics and limitation of the existing methods.

Methods	Characteristics	Limitations
[30]	Use of less space because building a tree with only the main lines from the sparse graph. fast access because it is constructed by using trees.	Increased DSTree construction time when the structure of the graph changes significantly. A large number of comparison operations occur because it detects frequent patterns after the full scan of DSTree.
[31]	Deployed at less cost than DSTree since DSMatrix is a two-dimensional array. Construct FP-Trees to detect frequent patterns and calculate the frequency of each pattern using the DFS.	Require a lot of computation and memory while constructing many FP-Trees for frequent pattern detection. DFS takes a long time to detect frequent patterns. Fail to resolve duplicate calculations, a problem caused by the use of sliding window techniques.
[32]	Fast and frequent patterns detection using simple AND computations. Significant frequent pattern detection based on connectivity.	Significant performance degradation because AND operations are performed on all patterns. Fail to resolve duplicate calculations, a problem caused by the use of sliding window techniques.

**Table 2:** Notations.

Notation	Description
DSMatrix	Matrix representing the occurrence of the edge as 1 or 0
$<v_{i},v_{j}>$	edge connecting the vertices $v_{i}$ and $v_{j}$
FiB	Number of occurrences of the edge in one batch processing
FiS	Total number of edges in the sliding window
slideNum	Value to calculate whether Edge will be frequent or not during future window slide
AND operation	Operations that determine whether two subgraphs occur simultaneously

**Table 3:** DSMatrix.

Edge	Contents (Batch1)	Contents (Batch2)	Contents (Batch3)
$<v_{1},v_{2}>$	1 1 1	1 1 1	1 1 1
$<v_{1},v_{3}>$	1 1 1	0 1 1	1 1 1
$<v_{1},v_{4}>$	1 0 0	1 1 0	0 1 0
$<v_{2},v_{4}>$	1 0 1	0 0 0	1 0 0
$<v_{3},v_{4}>$	1 0 1	0 1 0	1 1 1

**Table 4:** Frequent subgraph pattern management table.

Edge	slideNum	FiB (Batch1)	FiB (Batch2)	FiB (Batch3)	FiS
$<v_{1},v_{2}>$	1	3	3	3	9
$<v_{1},v_{3}>$	1	3	2	3	8
$<v_{1},v_{4}>$	0	1	2	1	4
$<v_{2},v_{4}>$	−1	2	0	1	3
$<v_{3},v_{4}>$	0	2	1	3	6

**Table 5:** Frequent subgraph pattern management table where patterns are added.

Subgraph	slideNum	FiB (Batch1)	FiB (Batch2)	FiB (Batch3)	FiS
$<v_{1},v_{2}>$	1	3	3	3	9
$<v_{1},v_{3}>$	1	3	2	3	8
$<v_{1},v_{4}>$	0	1	2	1	4
$<v_{2},v_{4}>$	−1	2	0	1	3
$<v_{3},v_{4}>$	0	2	1	3	6
$<v_{1},v_{2},v_{3}>$	1	3	2	3	8
$<v_{1},v_{3},v_{4}>$	0	2	1	3	6
$<v_{1},v_{2},v_{3},v_{4}>$	0	2	1	2	6

**Table 6:** DSMatrix after the window slide moves.

Edge	Contents (Batch2)	Contents (Batch3)	Contents (Batch4)
$<v_{1},v_{2}>$	1 1 1	1 1 1	111
$<v_{1},v_{3}>$	011	111	000
$<v_{1},v_{4}>$	110	010	111
$<v_{2},v_{4}>$	000	100	111
$<v_{3},v_{4}>$	010	111	010

**Table 7:** Frequent subgraph pattern detection after window slide moves.

Subgraph	slideNum	FiB (Batch2)	FiB (Batch3)	FiB (Batch4)	FiS
$<v_{1},v_{2}>$	1 → 0	3	3		
$<v_{1},v_{3}>$	1 → 0	2	3		
$<v_{1},v_{4}>$	0	2	1	3	6
$<v_{2},v_{4}>$	−1 → 0	0	1		
$<v_{3},v_{4}>$	0	1	3	1	5
$<v_{1},v_{2},v_{3}>$	1 → 0	2	3		
$<v_{1},v_{3},v_{4}>$	0	1	2	0	3
$<v_{1},v_{2},v_{3},v_{4}>$	0	2	1	0	3

**Table 8:** Performance evaluation environment.

Feature	Contents
Processor	Intel(R) Core(TM) i5-4440 3.10 GHz
Memory	8 GB
Disk	1 TB
Program language	Java
